# Supplementary material for: A Critical View of the Application of the APEX Software (Aqueous Photochemistry of Environmentally-Occurring Xenobiotics) to Predict Photoreaction Kinetics in Surface Freshwaters
Source: Molecules. 2019 Dec 18;25(1):9. doi: 10.3390/molecules25010009 (PMC7017383; doi:10.3390/molecules25010009)
Supplement: Supplementary file 1 [file molecules-25-00009-s001.zip › 3.2.4_gcc-4.4.0.zip!/3.2.4_gcc-4.4.0/3.2.4_gcc-4.4.0/doc/octave/html/interpreter/Debug-Mode.html]

Debug Mode - Untitled


Next: Call Stack,
Previous: Breakpoints,
Up: Debugging


---

### 13.4 Debug Mode

There are two additional support functions that allow the user to
interrogate where in the execution of a script Octave entered the debug
mode and to print the code in the script surrounding the point where
Octave entered debug mode.

— Loadable Function: **dbwhere** ()  
> Show where we are in the code
>
>
>
> **See also:** dbclear, dbstatus, dbstop.

— Loadable Function: **dbtype** ()  
> List script file with line numbers.
>
>
>
> **See also:** dbclear, dbstatus, dbstop.

You may also use `isdebugmode` to determine whether the debugger is
currently active.

— Command: **isdebugmode** ()  
> Return true if debug mode is on, otherwise false.
>
>
>
> **See also:** dbstack, dbclear, dbstop, dbstatus.

Debug mode also allows single line stepping through a function using
the commands `dbstep`.

— Command: **dbstep** n  
— Command: **dbstep** in  
— Command: **dbstep** out  
> In debugging mode, execute the next n lines of code. If n is
> omitted execute the next line of code. If the next line of code is itself
> defined in terms of an m-file remain in the existing function.
>
> Using `dbstep in` will cause execution of the next line to step into
> any m-files defined on the next line. Using `dbstep out` with cause
> execution to continue until the current function returns.
>
>
>
> **See also:** dbcont, dbquit.
